# Influence of Interleukin-8 and Neutrophil Extracellular Trap (NET) Formation in the Tumor Microenvironment: Is There a Pathogenic Role?

**DOI:** 10.1155/2019/6252138

**Published:** 2019-04-10

**Authors:** Manuela Gonzalez-Aparicio, Carlos Alfaro

**Affiliations:** ^1^Gene Therapy Program, Fundacion para la Investigacion Medica Aplicada, CIMA, Universidad de Navarra, Instituto de Investigación Sanitaria de Navarra (IdiSNA), Av. Pio XII 55, Pamplona 31008, Spain; ^2^Oncohematology Research Group, Navarrabiomed, Complejo Hospitalario de Navarra, Universidad Pública de Navarra, Instituto de Investigación Sanitaria de Navarra (IdiSNA), Irunlarrea 3. 31008 Pamplona, Spain

## Abstract

In this review, we will highlight several studies that revolve around interleukin-8 (IL-8) and show the multiple facets that could take in the tumor microenvironment. Chemokines that attract neutrophils (to a large extent, IL-8) can have a bimodal behavior inducing the migration of them in the first place and later favoring the formation of NETs in the place of emission focus of the chemokine. Also, this mechanism occurs when neutrophils migrate to tumor cells and where the extrusion of NETs in the tumor is observed. A possible participation of NETs in cancer progression was considered; however, until now, it is difficult to decide if NETosis plays a pro- or antitumor role, although it is necessary to emphasize that there is more experimentation focused on the protumorigenic aspect of the NETs. The formation of NETs has a relevant role in the inhibition of the immune response against the tumor generated by neutrophils and in turn favoring the processes involved in the development of tumor metastasis. It is striking that we do not have more complete information about the effects of circulating chemokines on neutrophils in cancer patients and hence the suitability of this review. No one has observed to date the impact that it could have on other cell populations to inhibit the arrival of neutrophils and the formation/elimination of NETs. However, the extent to which NETs affect the function of other cells of the immune system in the tumor context has not been directly demonstrated. It is necessary to identify possible combinations of immunotherapy that involve the modulation of neutrophil activity with other strategies (immunomodulatory antibodies or adoptive cell therapy). Therefore, knowing the mechanisms by which tumors take advantage of this ability of neutrophils to form NETs is very important in the search for antitumor therapies and thus be able to take advantage of the possible immunotherapeutic combinations that we currently have in clinical practice.

## 1. Characteristics and Effects of IL-8

Interleukin-8 (IL-8), also known as CXCL8, is a proinflammatory chemokine [1] of CXC type that is processed to give rise to a functionally competent protein of 77 amino acids in the case of IL-8 produced by parenchymal cells and 72 amino acids in the case of the one produced by monocytes and macrophages. The production of IL-8 is mainly regulated by NF-*κ*B transcription factors and in minor media by NF-IL6 [2]. IL-8 is a fundamental chemokine to promote tissue infiltration by polymorphonuclear leukocytes [3, 4]. This chemokine is not conserved between species since there is no homolog in the mouse genome that is difficult to study including their functions in murine models genetically transformed.

The biological effects of IL-8 are exerted through two surface receptors called CXCR1 and CXCR2 [5, 6]. These receptors share a remarkable similarity and homology in their sequence that suggests that they are the product of a gene duplication. The signals from these receptors are transmitted through the plasma membrane through conformational changes that expose regions in the intracellular loops of the receptor. These conformational changes allow G proteins to bind (mainly G*α*i, although possibly other G proteins insensitive to pertussis toxin are also involved) [7]. Activation of G proteins determines the activation of PI3Kinase, phospholipase C, and members of the RAS family [8]. These events in turn determine the activation of the AKT-mTOR pathway, the activation of PKC, and the entry of ionic calcium into the cytosol [9]. The reorganization of the cytoskeleton is mainly mediated by Rho GTPases and the FAK kinase [10] that reorganizes via ARP 2/3 the actin cytoskeleton [9]. These signaling pathways can have effects on multiple leukocyte functions in addition to migration [11].

The chemoattraction of neutrophils to the inflammatory focus is mediated by different substances, among which a family of chemokines stand out those that act on the CXCR1 and CXCR2 receptors. The signals from these receptors are transmitted through the plasma membrane to G proteins (mainly G*α*i, although other G proteins insensitive to pertussis toxin are also possibly involved). Both CXCR1 and CXCR2 receptors do not share the same ligands. CXCR1 is activated only in response to CXCL1, CXCL6, and CXCL8, while CXCR2 is activated by several CXC chemokines, in addition to the aforementioned, such as GRO*α*, GRO*β*, the neutrophil-activating peptide GPC-2, NAP-2, and ENA-78. The exposure of these receptors to their ligands determines the intracellular internalization and therefore the desensitization of the cell to the chemokine [12]. In addition, the functions of CXCR1 and CXCR2 do not overlap, since the first in addition to chemotaxis seems to play an important role in the activation of the microbicidal capacity of polymorphonuclear leukocytes [13].

The expression of receptors for IL-8 in cancer cells, endothelial cells, and tumor-associated macrophages suggests that the secretion of IL-8 by cancer cells should have an intense effect on the tumor microenvironment [9, 14]. IL-8 determines in endothelial cells proangiogenic effects that include the proliferation, survival, and migration of vascular endothelial cells [15]. It is also thought that IL-8 has beneficial autocrine and paracrine effects for the tumor cells themselves [9, 14].

The effects of IL-8 in leukocyte populations of cancer patients are not well known. It is possible that they regulate the entry into the malignant tissue of myeloid populations. We have shown that IL-8 attracts and retains dendritic cells specialized in inducing T lymphocyte responses [16]. We have also seen that IL-8 produced by xenografted human tumors in mice determines the disorientation of the migration pattern of human dendritic cells without affecting their immunogenic capacity [17]. It is striking that we do not have information about the effects of circulating IL-8 on neutrophils in cancer patients and hence the suitability of this review.

The development of anti-IL8 humanized monoclonal antibodies, such as ABX-IL-8, has allowed at studying the effect of suppressing IL-8 signaling in tumor progression [18]. Thus, it has been seen that the administration of ABX-IL-8 to mice carrying xenografts of bladder cancer decreases their tendency to metastasize and progress [18], as also happens in similar models of melanoma and prostate cancer [19]. Recently, it has been documented that IL-8, through its proangiogenic effects, is implicated in resistance to VEGF inhibitor drugs such as sunitinib or bevacizumab [20].

It is important to keep in mind not only the effects of IL-8 in the tumor microenvironment, since we must not forget the chemotactic effects on the innate response mediated by circulating leukocytes against infections. It is well known that cancer patients have a higher incidence of infections by pyogenic and fungal bacteria [21]. Some cases can be explained by neutropenia secondary to myelosuppression by different chemotherapeutic agents [22]. However, in cases with normal leukocyte levels in the peripheral blood, there is also a marked tendency to infectious processes that are frequently serious. It is possible that elevated levels of IL-8 disorient the migration of polymorphonuclear leukocytes and make it difficult for them to follow the gradient of IL-8 to migrate to sites of acute infection [23]. If this is correct, it can be explained that the plasma concentration of IL-8 will determine, at least, a certain propensity to develop acute infections and/or to increase its severity and duration.

## 2. IL-8 Action on Polymorphonuclear Cells

The interaction of cells expressing specific receptors for a specific chemokine with chemokine agonist determines two molecular consequences: (1) polarization and cell migration towards the chemokine concentration gradient and (2) internalization of specific receptors for that chemokine with the consequent desensitization of the capacity to respond to it [24]. In the case of IL-8, the receptors that are stimulated and desensitized are CXCR1 and CXCR2 [11, 25].

Chronic and continued exposure of neutrophils, or other strains of leukocytes and their myeloid hematopoietic precursors at high concentrations of IL-8, determines the functional desensitization of CXCR1 and CXCR2 receptors, or at least a disorientation in their chemotactical migration [4]. By disorientation in its migration, the high concentration of IL-8 in the whole organism determines a disruption of the concentration gradients that guide the chemotactic movement. These phenomena can occur in the organism of patients with advanced cancer, and it has a consequence that peripheral polymorphonuclear leukocytes will migrate with lower efficacy towards IL-8 gradients. Therefore, extravasation to infected/inflamed tissues will occur with less efficiency and accordingly, susceptibility to bacterial infections and their severity will be greater. Chemotherapy often determines neutropenia and therefore aggravates this situation if the migration to form pus is qualitatively altered.

An important role of IL-8 is the attraction of multiple lymphocyte populations to the same source of emission [26]. It is especially important in the regulation of the immune response for tumor development and may even be responsible in part for the suppression of this antitumor response [27, 28].

In this case, we have verified that IL-8 is able to attract both DC and neutrophils to the same place, where they are in close contact [17]. This allows a transfer of material between the cells that subsequently can trigger an immune response that favors tumor development.

Our experiments make evident complex relationship between PMN and DC. Physiologically, the PMNs are much more numerous than the DC and, therefore, could act as possible accumulators of antigens and microbial molecules for DC. DC can internalize the material present in the PMN and then modulate DC functions while transferring the antigens that PMN may carry [17]. It is within the possibility that this phenomenon may occur in the same manner by endogenous DC and could take special importance in the immune response against the tumor. However, exactly how relevant are these functions for the overall physiology of the immune system still remains to be seen.

## 3. NET Formation and Implications

The process of NET generation, also called NETosis, is a specific type of cell death, different from necrosis and apoptosis [29, 30]. NETs are formed by neutrophils upon contact with several bacteria or fungi as well as with activated platelets or under the influence of numerous inflammatory stimuli, and this process is associated with dramatic changes in the morphology of the cells [31]. The main components of NETs are DNA and granular antimicrobial proteins that determine their antimicrobial properties. Recent studies have shown that neutrophils are able to perform beneficial suicide to create a sophisticated and unique microbicide network composed of cellular content linked to the chromatic frame [32, 33]. The pathogens strapped in these NETs are killed by oxidative and nonoxidative mechanisms [30, 34].

Therefore, it is a powerful tool that primary serves as a protector from severe infections, but this effective defense tool is also a double-edged sword in the immunity [35, 36]. For this reason, overproduced NETs could provoke coagulation disorders, certain autoimmune diseases, and even cancer metastases [37].

On the other hand, several studies have discovered that chromatin and proteases released in the circulatory system during NET formation can regulate procoagulant and prothrombotic factors [34]. In the same way, they could take part in clot formation in blood vessels and might be cytotoxic for tumoral cells [38]. It is speculated that NET components like myeloperoxidase, proteinases, and histones possess antitumorigenic effects by means of actual killing of tumor cells. Therefore, its main function would be to inhibit their growth, activate the immune system, or scaffold directly tumor cells, preventing in this way their further dissemination. Furthermore, probably through histones, NETs can kill activated endothelial cells thus damaging tumor-feeding blood vessels [39, 40].

Alternatively, NETs which harbor potent proteases could be protumorigenic by degradation of the extracellular matrix [41]. So these structures would be able to promote extravasation and metastasis besides helping metastatic cells to evade the immune response as by forming a barrier between cancer cells and the immune system [42]. In this manner, NETs could help cancer cells to escape immune recognition.

Therefore, it is important to increase the knowledge about paths underlying NET formation and degradation processes if we want to efficiently fight with bacterial infections and certain diseases, as in cancerous processes [29, 43].

## 4. Polymorphonuclear Leukocytes and NET Production in Tumor Microenvironment

Neutrophils are the most abundant leukocyte type of the peripheral blood and play a crucial role in the defense against microorganisms [44]. Neutrophils are rapidly recruited to foci of acute inflammation where their main role is to induce the death of bacteria and fungi [45]. As we have commented previously, the microbicidal mechanisms that neutrophils use are mainly phagocytosis, degranulation of enzymes and bactericidal cationic peptides, and production of free oxygen radicals, as well as the capacity to protrude their nuclear DNA by forming networks [46–48].

Several groups demonstrate desensitization of IL-8-induced migration of polymorphonuclear leukocytes from healthy individuals and cancer patients after preincubation with IL-8, while respecting migration to other stimulus chemistries such as E. coli bacteria irradiated with ultraviolet light [49]. Likewise, it has been verified that the exposure to IL-8 determines the internalization and decrease of the surface expression of CXCR1 and CXCR2, as can be seen by flow cytometry [49, 50]. Finally, numerous groups confirmed the presence of high circulating levels of IL-8 in a series of patients with advanced neoplasms [51, 52]. In this context, elevated serum concentrations of IL-8 are observed in patients with advanced cancer that are not observed in healthy volunteers [51]. It is well known that the synthesis of IL-8 is very abundant in human tumor cell lines both *in vitro* and *in vivo* [53–55].

IL-8 for its role in attracting polymorphonuclear leukocytes has a direct and indirect role in the stimulation of angiogenesis [25]. Genetic studies to clarify the role of IL-8 in cancer are complex since IL-8 is not conserved in rodents, and, for this reason, studies in transgenic or knockout mice cannot be performed. In addition, studies with IL-8 in tumor xenografts are difficult to interpret because they lack specific receptors for IL-8 in both leukocytes and endothelial cells of the human tumor-carrying mouse, although IL-8 exerts some activity on the mouse CXCR1 receptor [56].

As we have previously commented, the expression of IL-8 is frequent in human tumors and its plasma concentration in most cases correlates directly with the tumor size [51, 52]. In turn, we have been able to demonstrate the biological effects of IL-8 in the repression of the antitumor immune response. These pathogenic functions include disorientation in the migration of dendritic cells or the attraction of suppressive myeloid cells [49, 57]. In turn, we have shown that IL-8 induces NETs in granulocytic MDSCs in the same way that it induces them on neutrophils [57, 58].

The role of neutrophils in the evolution of cancer is not known in depth. Massive expression analyses using TCGA have associated a genetic signature of the presence of polymorphonuclear leukocytes with an adverse prognosis in the development of the disease in several types of cancer [59]. Many studies suggest that neutrophils may acquire immunoregulatory capabilities by acquiring the expression of molecules such as arginase-1 that inhibit the T-lymphocyte-mediated immune response [60, 61]. In fact, a subpopulation of immature neutrophils has been found to be abundant in cancer patients and mice, which is called granulocytic myeloid-derived suppressor cells (Gr-MDSC) [62].

Preliminary data have demonstrated that numerous chemoattractant stimuli tested (CXCL1-8, LTb4, and formyl peptides) to date are able to induce the extrusion of NETs in neutrophils at high concentrations (Dr. A. Teijeira, personal communication, June 15, 2018). Neutrophils are able to polarize and migrate towards foci of tumor cells that express these chemotactic factors in abundance [63, 64]. Upon reaching the maximum production zone of the chemokine, the gradient of chemotactic concentration disappears. It is possible that it is upon reaching a high level of receptor occupancy when the chemotactic stimulus determines the extrusion of the DNA and the formation of NETs [65]. By means of intravital microscopy in tumors, previous studies observed that neutrophils present directional motility to the tumor and the formation of DNA NETs (personal communication). In the tumor context, these structures have been associated mainly with processes that favor metastasis [66–68]. An intravascular role of neutrophils is proposed, whose DNA favors the persistence and survival of tumor cells in the bloodstream [69, 70]. A recent study in mice also suggests that NETs favor the invasive capacity of tumor cells favoring their migration [71]. However, the extent to which NETs affect the function of other cells of the immune system in the tumor context has not been directly demonstrated.

## 5. IL-8 Derived from Tumors Contributes to the Chemotactic Recruitment of Myeloid-Derived Suppressor Cells

We have explored the relevance of the IL-8 attraction influence towards possible suppressive populations that are found in the tumor microenvironment [57].

The suppressive myeloid cells (myeloid-derived suppressor cells (MDSC)) constitute a heterogeneous population of immature cells composed of macrophages, granulocytes, and other populations of myeloid origin in early stages of differentiation [72]. They especially have an important immunosuppressive component of T cells in cancer patients, as well as they are being able to promote the expansion of regulatory T cells [72, 73]. Currently, the factors capable of attracting this cellular subtype to the tumor microenvironment are poorly understood. We have verified in previous works [57] that IL-8 is a chemokine produced by cancer cells and whose serum concentration correlates with the tumor burden of patients and with a poor prognosis of the disease. We have shown that IL-8 produced by cancer cells attracts by chemotaxis to suppressive myeloid cells obtained from the peripheral blood of patients with advanced cancer and that this chemotactic activity can be interrupted pharmacologically in tests in mice [57]. Surprisingly, it was also found that IL-8 activates granulocytic myeloid suppressor cells to produce the formation of extracellular neutrophil traps (neutrophil extracellular traps (NETs)). These mechanisms mediated by IL-8 could be relevant in the establishment of a tumor microenvironment that favors the attraction of leukocytes that help the tumor to evade the immune system [74]. Definitely, IL-8 produced by tumors contributes to the chemotactic attraction of suppressive myeloid cells and their functional control [25, 57].

## 6. Importance of Work in Oncology

The development of humanized monoclonal antibodies against CXC chemokines (such as ABX-IL-8), as well as drugs that inhibit CXCR1/2 receptors, allowed us to study the effect of suppressing the signaling by IL-8 or other ligands of these receptors in the tumor progression [75–77]. Thus, it has been seen that the administration of ABX-IL-8 to mice carrying xenografts of bladder cancer decreases their tendency to metastasize and progress, as it also happens in similar models of melanoma and prostate cancer [18, 78].

A possible option in the treatment of cancer would be the combination of effective immunotherapy strategies with treatments that interfere with neutrophil chemoattraction and NET extrusion [79]. Therefore, knowing the mechanisms by which tumors take advantage of this ability of neutrophils to form NETs is important in the search for new antitumor therapies and possible therapeutic combinations.

For this reason, it is necessary to analyze the mechanism through which CXC chemokines functionally damage human polymorphonuclear leukocytes, analyzing the correlation between IL-8 and leukocyte migration parameters as well as the propensity to severe infections in patients. It would be possible in this way to reveal a determinant and potentially treatable factor in the pathogenesis of the susceptibility to metastasize in patients with advanced cancer.

## 7. Final Conclusions

We have observed the implication of IL-8 as a biomarker in several tumors and as a chemoattractant of neutrophils and human myeloid suppressor cells. In conclusion, there could be a much defined axis where IL-8 plays a very important role in the recruitment of certain lymphocyte populations and tumor development, including the way in which tumors are capable of developing metastasis. The influence of IL-8 is like an actor who has different roles in the same tumor movie.

Although it is still early to unravel the true role of NETs in the organism, it seems evident that an antimicrobial role is something innate for PMNs as the first defense mechanism. The problem lies in the particular use by certain cell types or the exacerbation of this production that could cause different pathologies or even favor certain metastatic events. Future research should focus on the possibility that tumor cells take advantage of DNA networks extruded by polymorphonuclear leukocytes and their immunosuppressive effect to metastasize successfully.
